# Brain Perfusion Impairment in Neurologically Asymptomatic Adult Patients with Sickle-Cell Disease Shown by Voxel-Based Analysis of SPECT Images

**DOI:** 10.3389/fneur.2013.00207

**Published:** 2013-12-20

**Authors:** Leonardo Deus-Silva, Leonardo Bonilha, Benito P. Damasceno, Andre L. F. Costa, Clarissa L. Yasuda, Fernando F. Costa, Allan O. Santos, Elba C. S. C. Etchebehere, Regis Oquendo-Nogueira, Renata Fockink, Claudio Fróes de Freitas, Edwaldo E. Camargo, Li M. Li, Fernando Cendes, Sara T. Saad

**Affiliations:** ^1^Department of Neurology, University of Campinas, Campinas, Brazil; ^2^Division of Neurology, Department of Neurosciences, Medical University of South Carolina, Charleston, SC, USA; ^3^Department of Orthodontics, City University of São Paulo, São Paulo, Brazil; ^4^Hematology and Hemotherapy Center, University of Campinas, Campinas, Brazil; ^5^Division of Nuclear Medicine, Department of Radiology, University of Campinas, Campinas, Brazil

**Keywords:** sickle-cell disease, brain perfusion, SPECT, voxel-based analysis

## Abstract

Cerebrovascular lesions are frequently observed in patients with sickle-cell disease (SCD) and these structural lesions are preceded by insidious perfusion deficits. Our aim was to investigate the presence of brain perfusion deficits in neurologically asymptomatic SCD patients, especially affecting microvessels. For this study, 42 SCD patients [33 sickle-cell anemia (HbSS), 6 sickle hemoglobin C disease (HbSC), and 3 sickle β-thalassemia disease (HbSβ)] with mean hematocrit of 25.1 (±4.85; 15.6–38.5) underwent brain perfusion single photon emission computerized tomography (SPECT) using the tracer ^99m^Tc-ECD. Images from SCD patients were compared to images of a healthy control group (29 females and 20 males, mean age 31 ± 8; range 25–49 years). Images underwent voxel-wise comparison of regional tracer uptake using paired *t*-test to estimate the probability of each voxel to have an increased or decreased tracer uptake. When compared to controls, SCD patients exhibited significantly reduced tracer uptake in basal ganglia and thalami, the anterior frontal region and the watershed region of the temporo-parietal-occipital transition (*p* < 0.05). Our study showed that neurologically asymptomatic adult SCD patients exhibit a pattern of reduced ^99m^Tc-ECD tracer uptake demonstrated by SPECT. Early diagnosis of this cerebral vasculopathy has prognostic implications and can be determinant in considering therapeutic alternatives to avoid increasing brain lesion load and progressive disability.

## Introduction

Severe hemolytic anemia, vascular occlusions, and cumulative organ damage secondary to infarcts are frequently found among the clinical manifestations of patients with sickle-cell disease (SCD). Neurological dysfunction is a well-known complication in patients with SCD. It is usually manifested as an acute cerebrovascular accident, or alternatively as a headache, seizure, or cognitive decline (1). Children and teenagers are particularly vulnerable to overt cerebrovascular complications of SCD (2). Steen et al. have shown that at an average age of 10 years, the estimated prevalence of vasculopathy in the homozygous for hemoglobin (Hb) S (SS) patients detected by magnetic resonance imaging (MRI) was 64% (3).

The Cooperative Study of Sickle-Cell Disease (CSSCD) demonstrated that the incidence rate of a first stroke by the age of 20 is 11% in SS patients (4, 5). While younger patients tended to have occlusive strokes, hemorrhagic strokes were more common in the 20–29 year old patients. The CSSCD also analyzed structural MRI from 312 patients, and reported that ischemic lesions were present in 13% of all SCD patients without a clinical history of overt stroke. These “silent infarcts” have been shown to be associated with neuropsychological decline, progressive brain injury, and are a significant risk factor for overt stroke in SCD patients (6, 7).

Currently, the screening for silent cerebrovascular disease in patients with SCD is performed by a combination of techniques including visual inspection of structural MRI, transcranial Doppler (TCD) and MR angiography (MRA) (8). A follow-up study by Seibert et al. (9) demonstrated that an abnormal MRA is highly predictive of stroke in asymptomatic patients with SCD, and suggested that screening for cerebrovascular disease should be performed first with TCD and followed by MRA if the TCD reveals any abnormality. However, this combination of techniques may occasionally fail to detect cerebrovascular abnormalities, especially at the microvascular level. Wang et al. (10) examined 78 asymptomatic children with SCD and observed that the structural MRI and TCD are often discordant, suggesting the need for a more sensitive and specific indicator of early cerebrovascular dysfunction. Indeed, TCD is not a reliable method to screen for silent infarcts. It is limited to the evaluation of cerebral blood flow (CBF) velocity of large arteries while silent infarcts are routinely found in patients with normal CBF velocity (10). Likewise, structural MRI may not be able to assess the status of the microcirculation where the sickled red blood cells obstruct the blood flow leading to regional ischemia and progressive organ injury without necessarily causing detectable structural abnormalities at the scale measured by this tool (11).

Perfusion and metabolic imaging techniques such as positron emission tomography (PET) have been used to investigate the microvascular blood flow in patients with SCD. Powars et al. (12) used PET to detect abnormalities missed by structural MRI in patients with SCD and a history of stroke and Reed et al. found a metabolic compromise that extended beyond any corresponding anatomic abnormality demonstrated on MRI (13). Perfusion MRI has also been used to assess CBF in asymptomatic SCD children with results depicting reduced perfusion in areas appearing to be normal on conventional MRI (14). Neurological complications due to microvascular compromise in SCD are still underestimated, and structural lesions are preceded by insidious perfusion deficits (15).

Single photon emission computerized tomography (SPECT) is a functional neuroimaging technique that allows a non-invasive evaluation of the physiological and pathophysiological status of the brain (16). Ethyl-cysteinate-dimer labeled with technetium-99 m (^99m^Tc-ECD) is a lipophilic compound that crosses the blood brain barrier, entering the neuronal cell where it is converted to a hydrophilic form, remaining trapped inside the cell. The distribution of this tracer in the brain is proportional to regional cerebral blood flow (rCBF). However, ^99m^Tc-ECD is a perfusion agent that demands a metabolic process of desertification inside the cell and is therefore an indirect indication of metabolism (15) PET imaging is characterized by a higher resolution, sensitivity and can be quantified in absolute units. However, SPECT is easier to perform as a routine procedure due to its lower cost, simplicity, and availability.

To investigate the hypothesis of an insidious brain perfusion compromise in SCD patients, a voxel-based analysis of ^99m^Tc-ECD SPECT images of the whole brain of neurologically asymptomatic adult patients with SCD was performed. Tracer uptake of SCD patients and tracer uptake in a group of neurologically healthy controls that did not suffer from SCD were compared to identify areas with abnormal perfusion.

## Materials and Methods

### Subjects and clinical assessment

Forty-two patients with SCD from the hemoglobinopathy outpatient clinics of the State University of Campinas Hematology and Hemotherapy Center (Hemocentro/UNICAMP), distributed as 27 females and 15 males with mean age of 33.4 years (± 10.55, range 18–60) were included in this study. The inclusion criteria were to be at least 18 years old, a clear clinical and laboratory diagnosis of any SCD and being available for regular follow-up. The laboratory diagnosis of SCD was based on Hb electrophoresis and genetic testing. The 42 patients had no neurological symptoms during the time of investigation. They were thoroughly examined by a neurologist upon their enrollment to exclude any unsuspected neurological disorder. They were submitted to a structural MRI of the brain to exclude any gross intracranial pathology. Systemic arterial hypertension, cancer, diabetes, vasculitis, pregnancy, AIDS, or any other relevant medical condition were exclusion criteria for this study.

The SCD types were distributed as follows: 33 HbSS (14 males and 19 females), 6 HbSC (1 male and 5 females), and 3 HbSβ (3 females). The mean steady state Hb level measured in the period of up to 2 weeks before or after imaging acquisition was 8.5 (±1.73; 5.2–13.5) and the mean hematocrit was 25.1 (±4.85; 15.6–38.5).

The group of patients with SCD was compared to a group of healthy control subjects (29 females and 20 males, mean age 31 ± 8; range 25–49 years) recruited within the local community, who were submitted to the same imaging protocol used in the SCD patients.

This study was approved by the Ethical Committee of our institution and informed consent was obtained from all patients.

### SPECT imaging

To perform brain perfusion SPECT, patients rested supine in a dark, quite room for 30 min before injection. To reduce brain stimuli, the patients had an intravenous catheter placed prior to tracer injection for the infusion of 0.9% saline solution at a slow rate. The patients were then injected with 1100 MBq of the rCBF SPECT tracer ^99m^Tc-ECD intravenously and remained at rest for 15–20 additional minutes. Image acquisition started 30 min post injection with a single headed scintillation camera (APEX SP6 ELSCINT, Haifa, Israel), equipped with a fan-beam collimator. Sixty views were acquired every 6° in a 360° fixed circular orbit around the patients’ heads (64 × 64 matrix, 30 s per view). Images were reconstructed by filtered backprojection and Metz filtering (FWHM = 14 mm). Attenuation correction was performed by Chang first order method (attenuation coefficient = 0.115 cm^−1^). Reconstructed transaxial images were reformatted and displayed as sagittal, axial, and coronal sections.

### Preprocessing of SPECT images

Reconstructed transaxial datasets were transformed into ANALYZE using MRIcro software (Chris Rorden^1^). Voxel-based analysis was performed using SPM2 (Wellcome Department of Cognitive Neurology^2^). Images were spatially normalized to a standard anatomical template using linear transforms followed by 16 non-linear iterations with medium regularization. After normalization, the images were convolved with an Isotropic Gaussian Kernel (FWHM) of 10 mm to minimize gyral inter-individual variability and create images that are more normally distributed thus permitting voxel-wise analysis. The normalized smoothed images underwent between group comparisons of regional tracer uptake by using paired *t*-tests. Contrasts were defined to estimate the probability of each voxel to have an increased or decreased tracer uptake in images from patients with SCD compared to images from the control group (17, 18).

### Statistical analysis

Group differences for age were assessed using one-way ANOVA, and the gender distribution was evaluated with the Chi square test.

The normalized and smoothed data was analyzed using SPM2. SPECT images from patients with SCD were compared to the SPECT images of normal controls using two-sample *t*-test. The ^99m^Tc-ECD uptake was standardized to the mean global uptake using proportional scaling. Only voxels with signal intensities above a gray matter threshold of 0.8 were entered in each analysis. The results from the analysis are a parametric map of *t*-statistic [SPM_(t)_] corrected for normal distribution [SPM_(z)_]. The statistical threshold was a false discovery rate (FDR) corrected *p* < 0.05 (19) with an extent threshold looking for clusters with at least 32 contiguous voxels (Height threshold: *T* = 2.67).

## Results

**Figure 1:**
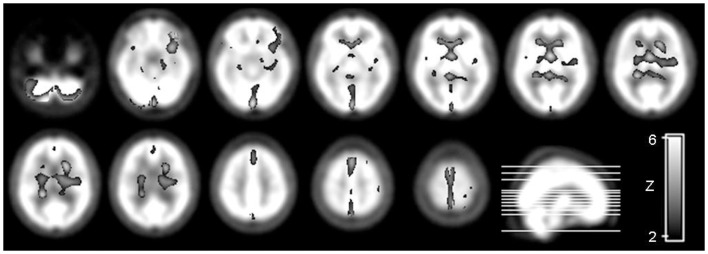
**Parametric map of the *t*-statistic [SPM_(t)_] depicts the location and the statistical significance of voxels with a significant probability of decreased tracer uptake and therefore reduced blood flow in patients with SCD compared to controls**. The map is illustrated in a “glass brain template” in radiological convention (i.e., the left side of the brain is displayed in the right side of the image). The statistical threshold was set at *p* < 0.05 looking for clusters with at least 32 contiguous voxels, corrected by multiple comparisons using FDR.

The values of the *t*-statistic [SPM_(t)_] and [SPM_(z)_] are displayed in Table 1, showing the regions of reduced tracer uptake in patients with SCD. Regions of tracer uptake excess in patients with SCD were not observed in comparison to controls. The three dimensional rendering of the parametric map of *t*-statistic was overlaid in a normal brain perfusion SPECT template as shown in Figure 1.

**Table 1:** **Results of the voxel-based comparison of SPECT images between controls and SCD patients**.

Structure	Cluster level analysis			Voxel level analysis				
	*p*-value (Bonferroni corrected for multiple comparisons)	Number of voxels within cluster	*p*-value (uncorrected for multiple comparisons)	*p*-value (FDR-corrected for multiple comparisons)	SPM_(t)_	SPM_(z)_	*p*-value (uncorrected for multiple comparisons)	Stereotaxic coordinates *x*, *y*, *z* (mm)
Right rostral cerebellar cortex	0.045	8485	0.013	>0.001	6.27	5.69	>0.0001	17, −80, −50
Medial occipital cortex				0.001	5.38	4.99	>0.001	−0, −86, −12
Left rostral cerebellar cortex				0.002	4.71	4.44	>0.0001	−18, −80, −50
Left frontal pole	0.271	3488	0.092	>0.001	5.84	5.36	>0.001	−42, 47, −17
Left ventral prefrontal cortex				0.001	5.58	5.15	>0.0001	−42, 39, −20
Left anterior ventral prefrontal cortex				0.001	5.09	4.75	>0.0001	−35, 44, −18
Left upper dorso-lateral fronto-parietal transition	0.021	10899	0.006	>0.001	5.77	5.3	>0.0001	−15, −39, 81
Upper medial fronto-parietal region				0.001	5.22	4.86	>0.001	−2, −36, 81
Left upper aspect of central sulcus				0.001	5.15	4.8	>0.0001	−15, −29, 81
Posterior horn of right lateral ventricle/temporo-parietal-occipital transition	0.001	21018	>0.001	0.001	5.3	4.93	>0.0001	21, −36, 20
Fornix/Central Forebrain				0.001	5.29	4.91	>0.001	−20, 0, 12
Left anterior frontal region				0.001	5.07	4.74	>0.001	−21, 18, 20
Upper dorsal aspect of left precentral gyrus	0.88	320	0.615	0.007	4.03	3.85	>0.0001	−44, −18, 66
Upper dorso-lateral aspect of left precentral gyrus				0.01	3.76	3.61	>0.0001	−48, −24, 63
Left superior parietal gyrus	0.954	34	0.897	0.013	3.55	3.42	>0.001	−30, −62, 68
Fourth ventricle/upper aspect of vermis	0.861	391	0.574	0.025	3.14	3.05	0.001	−2, −41, −24
Upper aspect of right temporopolar cortex	0.901	237	0.672	0.029	3.04	2.95	0.002	36, 15, −21
Left superior frontal sulcus	0.939	89	0.813	0.033	2.97	2.89	0.002	−27, 27, 56
Right insula	0.938	91	0.811	0.037	2.89	2.82	0.002	45, −3, 8

*The table outlines the anatomical locations where reduced ^99m^Tc-ECD tracer uptake was observed in patients with SCD. Each anatomical location is followed by the display of the corresponding *p*-values and the cluster size in voxels of the cluster of contiguous voxels that was observed to show reduced perfusion. Each location is also followed by the *t*-statistic [SPM_(t)_] corrected for normal distribution [SPM_(z)_] of the analysis performed with individual voxels. Finally, the stereotaxic coordinates for the anatomical locations is displayed. A Height threshold: *T* = 2.67, *p* = 0.004 (0.968), was used, with *p*-values adjusted for the number of voxels contained within the entire volume. We restricted the analysis to voxels that belonged to clusters with at least 32 voxels*.

A widespread reduced tracer uptake, corresponding to reduced blood flow was found in patients with SCD when compared to normal controls. In summary, we observed hypoperfusion involving the cerebellar hemispheres and vermis, the frontal and prefrontal cortex, especially the left, the fronto-parietal transition, the posterior periventricular region, the temporo-parietal-occipital transition, the central forebrain, and the ventral aspect of basal ganglia and thalami.

## Discussion

Our results demonstrate that neurologically asymptomatic adult SCD patients exhibit brain perfusion abnormalities in areas primarily supplied by small and terminal branches of arterial blood vessels. Specifically, we observed a decreased tracer uptake in the basal central forebrain, including the basal ganglia and thalami, watershed areas of the anterior circulation and cerebellar and occipital cortex. These findings may contribute to the understanding of mechanisms underlying the evolution of cerebral vasculopathy of SCD patients.

Silent cerebral vasculopathy in SCD patients is a major topic of research because it is strongly associated with increased stroke risk and progressive cognitive decline (20). Wang et al. (19) have recently demonstrated psychometric decline in performance with age in SCD patients with normal MRI, indicating brain dysfunction in SCD patients even when no structural damage is detected by MRI (21). A recent systematic review has shown that perfusion compromise precede the structural changes and TCD abnormalities usually found in SCD patients and therefore the assessment of cerebral blood perfusion can be of potential value in addressing brain abnormalities at the tissue level (22).

Significant hypoperfusion in the basal ganglia and thalami was found in the present study. These areas have already been described as frequent sites of ischemic damage in SCD patients. Pegelow et al. (23) evaluated 47 neurologically normal SCD children with MRI and found a total of 129 lesions; of these, 14 lesions were in the thalami and basal ganglia. In the CSSCD 27% of the patients with MRI abnormalities were found to have lesions in the thalamus and basal ganglia (23). Our results complement research showing the caudate, thalamus, and cortex as areas of selective vulnerability to damage in SCD patients (24). The hypoperfusion we observed in the thalamus and basal ganglia is possibly an additional factor accounting for the development of cognitive decline in SCD patients. Similar to vascular dementia (25), the slowness of mental labor, attention deficits, and dysfunction of working memory found in SCD patients could be attributed in part to disruption of thalamic function and interruption of thalamocortical connections.

We observed hypoperfusion in the frontal lobes, especially in medial areas. These regions are also recognized as frequently damaged in SCD patients. Studies using PET to evaluate brain perfusion in SCD patients have previously demonstrated hypometabolism in frontal areas and hypoperfusion in areas that appear to be normal on structural MRI (12, 26, 27). Pegelow et al. (28) performed a longitudinal assessment of MRI changes on the brains of SCD patients and observed that the frontal lobe was the most frequent location of ischemic damage in both neurologically asymptomatic and patients with a previous stroke (28). Frontal lobe lesions were detected in 81% of the children with silent infarcts and in 94.4% of those with a history of prior stroke. This finding suggests an early involvement of the frontal lobes in SCD brain vasculopathy and this may be an important predictive factor for overt stroke, since children with silent infarcts were significantly less likely to have lesions in the frontal cortex, as compared to patients with a history of a previous stroke. These findings are in accordance with the results of the work of Balci et al. (29) whom have found microstructural abnormalities of various brain areas in SCD patients using diffusion-tensor imaging.

We recognized low tracer uptake in cerebellum, interestingly affecting the cerebellar hemisphere contralateral to the most hypoperfused cortical hemisphere. This hypoperfusion may result from functional disconnection of the contralateral cerebellar neurons from their correspondent cerebral neurons. Interruption of the cerebropontine-cerebellar pathway is thought to be the most likely mechanism of this remote transneuronal metabolic depression known as diaschisis (30) and SPECT is a sensitive method for detecting this crossed cerebellar hypoperfusion (31).

Single photon emission computerized tomography imaging is a low-sensitivity method to white matter perfusion. The SPECT tracer distribution has a gray matter to white matter 4:1 ratio, and as a consequence cortical hypoperfusion is more accurately visualized. In this study we observed clusters of hypoperfused areas in patients with SCD that spanned over subcortical white matter, especially in the temporo-parietal-occipital transition. These findings are possibly a reflection of a widespread reduction of perfusion affecting the surrounding gray matter. These findings may improve our understanding of the progression of SCD brain vasculopathy. The observed reduction of tracer uptake may reflect ischemia, hypometabolism or both (15). Patients with SCD are chronically anemic and this condition increases the CBF due to hemodilution and as a compensatory response to decreased oxygen delivery (3). However, this hyperemic status predisposes to turbulent blood flow and subsequent endothelial cell injury, leading to hemodynamic shear stress and vessel wall damage (1, 32). Besides that, erythrocytes having HbS are very sensitive to oxygen saturation reduction. Polymerization of deoxygenated HbS is the initial step of a multifactorial, heterocellular event, causing erythrocyte sickling in the microvasculature and subsequent adhesion of these cells to endothelium which triggers a vast array of vascular microenvironmental modifications (33). This sickle-cell-induced vaso-occlusion is a very complex process and involves the participation of vascular cell adhesion molecules, especially VCAM-1, proinflammatory cytokines, leukocytes, and other active metabolites, all contributing to the enhanced cell-endothelium adherence (34).

Coupling of blood flow and metabolism is the most important control of the cerebral circulation. Neuronal activation increases the consumption of glucose and oxygen and therefore induces an increase in CBF in order to accommodate this metabolic demand (35). However, in SCD patients, these auto-regulatory mechanisms might be compromised due to progressive vascular damage secondary to repetitive sickling (1, 24). This auto-regulatory failure is even more significant in hypoxemic conditions, in which the expected cerebral vasodilation response may not occur as it might already be exhausted (36, 37). We speculate that the observed hypoperfusion results from a mixture of vaso-occlusion leading to ischemia, vascular auto-regulatory failure, and selective tissue damage secondary to an insufficient oxygen delivery in high metabolic states. A form of non-erythroid globin found in some areas of the brain (brain Hb) has been described as a source of oxygen in states of hypoxemia and may then act to provide a homeostatic mechanism in anoxic conditions of high metabolic areas. This mechanism could play a role in protecting the tissue at risk (38). As presumed to occur in some neurodegenerative diseases, we could also consider the possibility of a malfunctioning interaction linking brain Hb expression to mitochondrial function in SCD patients leading to neuronal damage or dysfunction. The hypoperfusion detected in the occipital lobes is an important illustration of damage in a region of high metabolism since visual stimuli are important determinants of neuronal activation.

Sickle-cell disease patients have a wide Hb range, from normal to severely anemic, and it is likely that the lower the level of Hb the more intense the hemolysis reflecting a more active sickling pattern and, therefore, more likely to influence brain perfusion and to provoke brain ischemia. However, it was not within the scope of our study to compare the abnormalities between groups with higher and lower levels of Hb, since our purpose was to analyze brain perfusion in SCD patients without neurological symptoms.

In conclusion, we observed an anatomical pattern of regional decreased brain perfusion in asymptomatic patients with SCD using ^99m^Tc-ECD SPECT. Specifically, we observed hypoperfusion in regions supplied by microvessels. We suggest that these brain areas may be more susceptible to insidious and silent brain injury due to SCD vasculopathy. These findings could have a prognostic application and offer a reliable measure for evaluating treatments designed to reduce the incidence of brain lesions and cognitive dysfunction in SCD patients.

## Conflict of Interest Statement

The authors declare that the research was conducted in the absence of any commercial or financial relationships that could be construed as a potential conflict of interest.

## Footnotes

^1^www.mricro.com

^2^http://www.ucl.ac.uk/ion/departments/repair/themes/cognitive
